# Females transplanted with ovaries subjected to hypoxic preconditioning show impair of ovarian function

**DOI:** 10.1186/1757-2215-7-34

**Published:** 2014-03-20

**Authors:** Luciana Lamarão Damous, Juliana Sanajotti Nakamuta, José Maria Soares-Jr, Gustavo Arantes Rosa Maciel, Ricardo dos Santos Simões, Edna Frasson de Souza Montero, José Eduardo Krieger, Edmund Chada Baracat

**Affiliations:** 1 Gynecology Division, Department of Obstetrics and Gynecology, Laboratory of Structural and Molecular Gynecology (LIM-58), Faculdade de Medicina da Universidade de São Paulo, São Paulo, Brazil; 2 Laboratory of Genetics and Molecular Cardiology, Heart Institute (Incor), Faculdade de Medicina da Universidade de São Paulo, São Paulo, Brazil; 3 Department of Surgery, Laboratory of Surgical Physiopathology (LIM-62), Faculdade de Medicina da Universidade de São Paulo, São Paulo, Brazil; 4 Galvão Bueno St, 499. Bloco A. Apto31. Liberdade, São Paulo 01506-000, SP, Brazil

**Keywords:** Ovarian transplantation, Hypoxic preconditioning, Apoptosis, Ovary, Rat

## Abstract

**Background:**

Cryopreservation of the ovarian tissue has shown promising results. However, there remain controversial issues such as the short half-life of grafts. In this aspect, there are some evidences that preconditioning the ovarian tissue before transplantation is beneficial.

**Objective:**

To determine the effect of hypoxic preconditioning in vitro on ovarian tissue prior to transplantation.

**Methods:**

Eighteen female adult Wistar rats, were sorted into three experimental groups. Ovaries were maintained in DMEM low glucose serum free at 37°C with 5% CO2, at atmospheric oxigen concentration (normoxia) or 1% O_2_ (hypoxia) for 16 hours. Oxigen concentration was determined by injection of nitrogen in the incubator. Animals submitted to ovarian transplantation immediately after oophorectomy were the Control Group (C). After this, the ovaries were implanted in the retroperitoneum with nonabsorbable suture and animals evaluated for thirty days after transplantation. Beginning on postoperative (PO) day 11, a daily collection of vaginal smear was carried out. Analyses comprised morphological, morphometric (counting ovarian follicles and corpora lutea) and immunohistochemistry for cleaved caspase-3 (apoptosis).

**Results:**

In normoxia and control groups all animals recovered their estrous cycles, while in the hypoxia group, two animals did not ovulate but, among those which did, resumption took longer than in the other groups (p < 0.05). The number of ovarian follicles and corpora lutea decreased significantly in the hypoxia group when compared to the other two groups (p < 0.001) and apoptosis was increased in the few ovarian follicles which remained viable (p < 0.001).

**Conclusion:**

The hypoxic preconditioning in vitro was not beneficial to the graft and worsened their viability, compromising its functionality or delaying the return of this.

## Background

Preservation of female fertility has become a main concern when evaluating first time patients at risk of becoming infertile or even sterile, especially in the case of children, adolescents and young women with cancer. This is necessarily a new challenge given the longer life expectancy of such patients as the direct result of advances in the diagnosis and treatment of the disease. Several assisted-reproduction techniques have been developed, the most recent of which is cryopreservation of ovarian tissue for subsequent transplantation [1-3].

Cryopreservation of the ovarian tissue has shown promising results. Besides not delaying the radiotherapy or chemotherapy treatment, for this technique can be carried out promptly, it is the only option available for children [1]. However, there remain a few controversial issues requiring experimental models for research on undesirable findings in patients who have undergone ovarian tissue transplantation, such as the comparatively short half-life of grafts, little responsiveness to hormone stimulation, and development of empty follicles [2-5].

Experimental studies of ovarian tissue transplantation generally exhibit a 50% to 90% reduction in follicle density [6,7], undesirable follicle activation (increase in the number of follicles) [8,9], and asynchronous growth of oocytes and follicle cells [10].

There are some evidences that preconditioning the ovarian tissue before transplantation is also beneficial. In a study of xenotransplantation, Lan et al. [11] demonstrated that the 6-hour fetal ovary culture without hypoxia prior to transplantation improved graft viability and increased the proportion of primordial and preantral ovarian follicles. Previous studies of ours showed that ischemic preconditioning of the ovary improves graft quality [12,13]. This motivated us to research other preconditioning forms to determine whether they could also increase the viability and longevity of grafts.

In vitro hypoxia has shown a number of beneficial effects, including cell lesion reduction, increased Vascular Endothelial Growth Factor (VEGF) expression with a resultant boost in angiogenesis, reduction of oxidative stress in endothelial cells [14], and an extension of the life span of stem cells derived from adipose tissue [15]. Our study set out to determine the effect of in vitro hypoxia on ovarian tissue prior to transplantation not only for the before mentioned reason but with a view to improving ovarian morphology and diminishing the apoptosis of ovarian follicle cells.

## Methods

Before being carried out, this project was submitted to the Ethics Committee, Department of Obstetrics and Gynecology and to the Committee for Analysis of Research Projects, University of São Paulo Medical School (CAPPesq-FMUSP – protocol 190/10). The project was carried out at the Medical Research Laboratory (LIM-58), Gynecology Discipline, Department of Obstetrics and Gynecology, FMUSP, with the cooperation of Laboratory Genetics and Molecular Cardiology, Heart Institute (Incor), FMUSP. The experimental procedures followed institutional guidelines for care and use of laboratory animals.

The study sample consisted of 18 virgin adult female Wistar (*Rattus norvegicus albinus)* rats weighing 200 to 250 g. The animals had access to a breed-specific food formula and water *ad libitum* throughout the experiment and were kept under adequate sanitary, lighting, and temperature conditions in the animal laboratory at the Heart Institute (Incor).

The animals were allocated to three study groups of six animals each. The groups were formed according to the following procedures: (1) Control: ovarian transplant immediately after oophorectomy; (2) Normoxia: between oophorectomy and transplantation, the ovaries remained overnight (16 h) in DMEM low glucose serum free at 37°C with 5% CO_2_, at atmospheric oxygen concentration (21% O_2_); (3) Hypoxia: the ovaries remained overnight (16 h) in DMEM low glucose serum free at 37°C with 5% CO_2_ and 1% O_2_ before transplantation.

Before the surgical procedures, vaginal smears were obtained daily from each rat at 8:00–9:00 every morning. Only those showing at least two consecutive normal 4-day vaginal estrus cycles were included in the experiment. The ovarian transplant was performed during the diestrous phase, and euthanasia during estrus.

The vaginal smear was obtained with a swab soaked in physiological solution and placed on a standard slide and immediately fixed in absolute alcohol for staining using the Shorr-Harris technique [16].

The slides were analyzed under a light microscope at 10x and 40x magnification. Based on the proportion of cells found in the smears, the estrous cycle phases were characterized as follows: (1) Proestrus, predominance of nucleated epithelial cells; (2) Estrus, predominance of anucleated, keratinized cells; (3) Diestrus, the same proportion of leukocytes and nucleated, keratinized epithelial cells [16].

The animals were anesthetized with intraperitoneally administered xylazine at a dose of 15 mg.kg^-1^ of body weight and ketamine at a dose of 60 mg.kg^-1^ of body weight. After the opening of the abdominopelvic cavity, the ovaries were identified; their pedicles were clamped and immediately ligated with 4–0 nylon suture. Next, the ovaries were removed bilaterally at the junction of the uterine horns and hemostasis was checked; the excised ovaries were then washed in physiological solution (0.9% NaCl). Finally, the fallopian tubes were resected with the periovarian adipose tissue fragments.

Each animal received a pair of autologous ovary transplants. The procedure was carried out with the aid of a surgical microscope (16×). In the control group, the ovaries were transplanted immediately. In the normoxia and hypoxia groups, the ovaries were placed in low-glucose DMEM (Dulbecco’s modified Eagle’s medium) in P35 Petri dishes, which were then incubated at 37°C overnight (16 h) in previously programmed concentrations of 5% CO_2_ and 21% O_2_ for the normoxia group and 5% CO_2_ and 1% O_2_ for the hypoxia group.

Afterwards, with a simple stitch of 4–0 nylon sutures, the intact ovaries were implanted in the retroperitoneum in the proximity of the great vessels (aorta and vena cava), each on one side of the psoas muscle, without vascular anastomosis. The wall closure was performed with a 5–0 nylon monofilament thread on two planes: (1) peritoneum – aponeurotic muscle and (2) skin.

Beginning on postoperative (PO) day 11, vaginal smears were obtained daily from each rat at 8:00–9:00 every morning until euthanasia, which was performed between day 30 and 35, with the rats always in estrus. The animals not in estrus during this period of time were excluded from the study.

The ovarian graft was removed and immediately fixed in 10% buffered formalin for 24 h for subsequent routine histological processing and hematoxylin-eosin staining. The ovarian cortex was sectioned into two pieces of equal size. After this procedure, the animals were euthanized with a lethal dose of the previously used anesthetics.

Morphological evaluation was achieved through descriptive analyses of the grafts. Assessment of follicular quality was based on cell density, the presence or absence of pyknotic bodies, and the integrity of the basement membrane and of the oocyte. According to these criteria, follicles were classified as normal or degenerated; only the former were characterized and quantified [17].

The viable follicles were classified as follows: (1) Primordial follicle, exhibiting only an oocyte and a layer of squamous cells; (2) Primary follicle, exhibiting an oocyte and a layer or more of cuboidal or prismatic cells but no antrum; (3) Secondary follicle, exhibiting an oocyte and an antrum. The mature follicle was that which contained an oocyte with a voluminous antrum. The corpus luteum was that which had intact luteal cells containing a voluminous nucleus and surrounded by capillary blood vessels [9,18,19].

The mature follicles were analyzed morphometrically in a 500-μm^2^ area [20]. To carry out the analysis, the follicles were classified into different stages forming three groups as follows: (1) developing follicles (primordial, primary, and preantral); (2) mature follicles (with only one voluminous antrum); (3) corpora lutea [18,21].

Images of the sections were obtained using an image acquisition software system (Leica DM2500); measurements were obtained using the Leica QWin V3 software. Counting was always done in four fields per section at 50× magnification.

Antibodies in the ovarian tissue were detected by the immunohistochemical-peroxidase method. Three-μm-thick sections were cut on silanized slides and then the following protocol was put in place: (1) Hydration: the sections were dewaxed with hot xylol (65°C) for 15 min and with cold xylol for another 15 min, washed in 95% absolute alcohol, and hydrated in deionized running water; (2) Antigen recovery: the sections were exposed to high temperatures in a pressure cooker with citrate buffer (pH6) at 125°C for 1 min; the sections were then washed in Phosphate Buffered Saline (PBS); (3) Blockage: endogenous peroxidase activity blocking was performed 10 times for 5 min each time with 3% 10v hydrogen peroxide (H_2_O_2_) and was followed by washing in water and PBS; (4) Incubation with the primary antibody: caspase-3 (caspase-3 p20 N-19:sc- 1226, Santa Cruz *Biotechnology)* (1:100 dilution) was diluted in BSA and applied on the sections and on the positive and negative tissue controls; the sections were then incubated overnight.

The sections were washed in PBS and incubated in the Novocastra Novolink Max (Polymer), RE7260-K, (Leica Microsystems, Wetzlar, Germany): both in the post primary block and in the polymer at room temperature for 30 min each. Subsequently, the sections were developed using the liquid 3,3’-diaminobenzidine (DAB) chromogen for 5 min. Next, the sections were washed in abundant running water and counterstained with Harris’ hematoxylin solution (Merck, Darmstadt, Germany). Immediately afterwards, they were washed in running water, dehydrated, cleared, and mounted using the resin-based mounting medium Entellan (Merck, Darmstadt, Germany).

Images of the sections were obtained using an image acquisition software system (Leica DM2500); measurements were obtained using the Leica QWin V3 software. Apoptosis assessment by means of caspase-3 was undertaken always using the percentage of positive area in the ovarian follicles in 4 different fields per animal at 200× magnification. Positively stained follicle cells were quantified as the percentage of positive cells per area. Negative control has no primary antibody. Two independent investigators blinded to the experimental treatments performed all measurements.

Results were expressed as means ± standard deviation of mean. One-way analysis of variance (ANOVA) with Tukey’s post-hoc test was utilized to compare groups. All statistical analyses were performed using Graphpad Prism 4.0 (Graphpad software Inc, CA, USA). P values of <0.05 were considered significant.

## Results

### Estrous cycle

**Figure 1:**
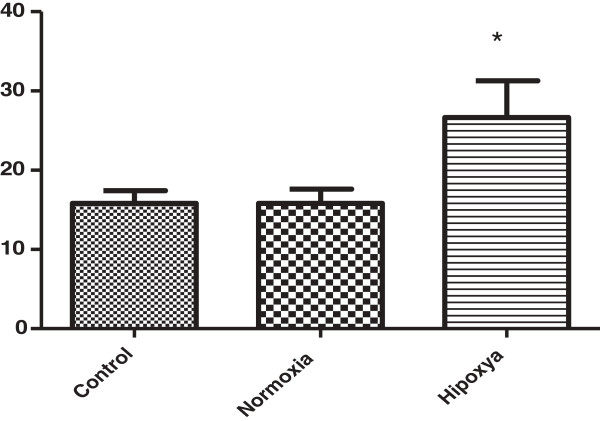
**Graphic representation of the resumption of the estrous cycle, in days.** *p < 0.05.

Analysis of the estrous cycle showed that all animals in the control and normoxia groups resumed regular estrous cycles with estrus returning within an average of 15.8 ± 3.63 and 15.8 ± 4.02 postoperative days, respectively. In the hypoxia group, two animals did not ovulate, but, among those which did, resumption took longer than in the other groups, an average of 26.7 ± 8.02 postoperative days (p < 0.05; Figure 1).

### Morphology

**Figure 2:**
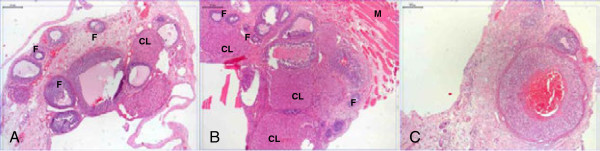
**Photomicrography of the ovaries after 30 days of transplantation 50×. (A)** Control Group; **(B)** Normoxia Group; **(C)** Hypoxia Group. F = Follicle; CL = Corpora Lutea; M = Muscle.

Ovarian graft morphology of the control and normoxia groups revealed ovarian follicles in diverse developmental stages and corpora lutea in all of the animals which were assessed (n = 12). The grafts subjected to hypoxia showed engorgement of numberless blood vessels and atypical ovarian follicles. Some of these had a reduced number of follicle cell layers infiltrated by leukocytes, pointing to a degenerative process; others had a blood vessels among the follicle cells; and a few had no oocytes, forming cysts in the ovarian stroma. In the latter group, there was reduced number of corpora lutea, most of which exhibited cells with pycnotic nuclei and leukocyte infiltrate, indicative of cell degeneration (n = 6) (Figure 2).

### Morphometry

**Figure 3:**
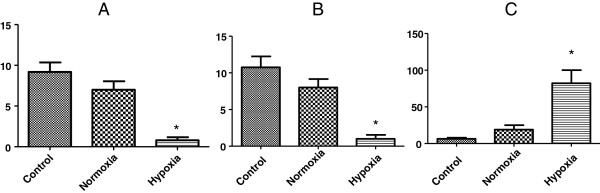
**Quantitative analyses of the ovaries after 30 days of transplantation (A) Number of mature ovarian follicles; B) Number of corpora lutea; C) Cleaved caspase-3 immunoreactivity.** *p<0.05.

The number of ovarian follicles and corpora lutea decreased significantly in the hypoxia group when compared to the other two groups (p < 0.001). These showed no significant difference between them (p > 0.05). Ovarian follicles: Control:9.2 ± 2.58; Normoxia: 7 ± 2.34; Hypoxia: 0.8 ± 0.83. Corpora lutea: Control: 10.75 ± 2.98; Normoxia: 8 ± 2; Hypoxia: 1 ± 1.22 (Figure 3A and B).

### Immunohistochemistry

**Figure 4:**
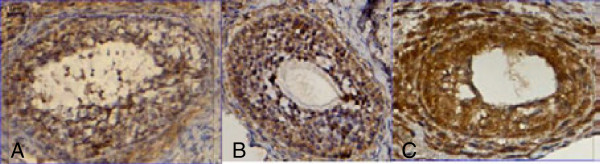
**Photomicrography of cleaved-caspase-3 immunoreactivity.** 400×. **(A)** Control Group; **(B)** Normoxia Group; **(C)** Hypoxia Group.

Hypoxia prior to transplantation boosted cleaved caspase-3 expression significantly in the few ovarian follicles which remained viable (p < 0.001), whereas apoptosis was similar in the normoxia and control groups (p > 0.05) (Control: 6.46 ± 4.48; Normoxia: 18.91 ± 13.8; Hypoxia: 82.13 ± 39.96) (Figures 3C and 4).

## Discussion

Transplantation of the cryopreserved ovary still needs further experimental studies, despite the promising results in the human reproduction field. At present, there are reports of several cases of autologous ovary transplants in orthotopic sites enabling spontaneous pregnancies [3]. The first case of spontaneous pregnancy was reported by Donnez et al. [22]. However, there are many reports on failures, primarily having to do with the short life span of the graft and with the absence of pregnancy even under hormonal stimulation. What might justify such failures is ischemia and fibrosis, both of which follow a transplant and result in the loss of the follicle population, preventing the recovery of fertility [23,24]. There are several experimental treatments for improving graft quality and one of the proposal is tissue cultivation [11].

Hypoxia preconditioning has yielded relevant results in the transplantation of other organs, such as the liver [25] and kidneys [26], and in cardiac regeneration after infarction [27]. In cell culture, hypoxia can increase proliferation rates and enhance differentiation along the different mesenchymal lineages [28]. Hypoxic preconditioning had a protective effect against mesenchymal stem cell apoptosis induced by hypoxia and reoxygenation via stabilization of mitochondrial membrane potential and upregulation of Bcl-2 and VEGF [29]. However, our results did not show any improvement in transplantation when the ovary was previously subjected to in vitro hypoxia.

Our study was based on the assumption that an in vitro hypoxia preconditioned ovary would respond better than an ovary exposed to hypoxia following transplantation, because it would have released environmental growth factors and free radicals, thus inducing early neoangiogenesis [30-33], which is crucial for preservation of the follicle pool. Our data, however, showed that overnight in vitro hypoxia before transplantation compromised graft viability with pronounced reduction in the number of viable ovarian follicles and functional corpora lutea.

The overall results were corroborated by cytological analysis of the vaginal smears, which showed a delayed resumption of the estrous cycle in the animals subjected to hypoxia as well as in those which remained in anestrus throughout the observation period (30–35 postoperative days). The option for cytology of vaginal smears was based on its great replicability. Other methods, such as hormone measurements, which are subject to variations induced by natural animal diversity or by environmental stress or handling, could have led to inconclusive results.

Lan et al. [11] were the first to assess the impact of in vitro ovary culture on follicle viability and development as well as the ideal length of culture time prior to xenotransplantation (0, 3, 6 or 9 days). The authors observed an increase in mature follicles in the ovaries that remained for 6 days in culture in a standard oven. This is the reason we chose this treatment in our study.

Several experimental hypoxia models for investigating pathophysiology or for testing different therapeutic strategies are described in the literature, both in vivo (liver) [34] and in vitro (cerebral cortex [35] and cardiac cells [36]). Wang et al. [36] investigated adult rat cardiac microvascular endothelial cells and Merino et al. [35] cortical neurons, both using 94% N_2_/5% CO_2_/1% O_2_. In this study, in order to induce hypoxia, we used the same gradient described in studies of other tissues [35-37], since we did not find any reports on in vitro hypoxia models for the ovary. However, the condition of severe hypoxia induced harmful changes in the graft, compromising its functional return. Further studies may help to clarify whether other oxygen concentrations (5% or 10%), as well as different hypoxia times, may produce a beneficial effect. We tested the 16 h-period, but smaller or even intermittent periods of time using the same gradient (1% O_2_) may turn out to be viable for organ preconditioning.

Although hypoxia before transplantation had bad effects on graft survival, culture in a standard oxygen gradient did not show to be detrimental to the grafts. In fact, our results were no better than those of the grafts transplanted without prior immersion in culture. Therefore, further studies of ovarian tissue culture for subsequent transplantation are needed.

## Conclusion

The hypoxic preconditioning in vitro on ovarian tissue prior to transplantation was not beneficial to the graft and worsened their viability, compromising its functionality or delaying the return of this in all parameters analyzed.

## Competing interests

The authors declare that they have no competing interests.

## Authors’ contributions

LLD conceived and designed study, performed experiments, interpreted data and wrote the manuscript. ECB and JEK conceived and designed study, corrected the manuscript and participated in substantial contribution to conception and revising it critically for important intellectual content. JSN performed experiments and wrote specific sections of this manuscript. RSS performed experiments and interpreted morphological data. JMS and GARM interpreted data and corrected the manuscript. EFSM designed study and participated in revising the manuscript. All authors read and approved the final manuscript.

## Authors’ information

Where the work was performed: Laboratory of Structural and Molecular Gynecology (LIM-58)/Department of Obstetrics and Gynecology and Laboratory of Genetics and Molecular Cardiology/Heart Institute (Incor), Faculdade de Medicina da Universidade de São Paulo, São Paulo, Brazil.
